# Apolipoprotein A-I and Cancer

**DOI:** 10.3389/fphar.2015.00265

**Published:** 2015-11-12

**Authors:** Maryam Zamanian-Daryoush, Joseph A. DiDonato

**Affiliations:** Department of Cellular and Molecular Medicine, and Center for Cardiovascular Diagnostics and Prevention, Cleveland Clinic, ClevelandOH, USA

**Keywords:** macrophage, apoA-I, HDL, cancer, cholesterol homeostasis, peptide mimetics, cancer therapeutic

## Abstract

High-density lipoprotein (HDL) and apolipoprotein A-I (apoA-I), the predominant protein in plasma HDL, have long been the focus of intense studies in the field of atherosclerosis and cardiovascular disease. ApoA-I, in large part, is responsible for HDL assembly and its main atheroprotective function, that of shuttling excess cholesterol from peripheral tissues to the liver for excretion (reverse cholesterol transport). Recently, a protective role for HDL in cancer was suggested from several large clinical studies where an inverse relationship between plasma HDL-cholesterol (HDL-C) levels and risk of developing cancer was noted. This notion has now been tested and found to be supported in mouse tumor studies, where increasing levels of apoA-I/HDL were discovered to protect against tumor development and provision of human apoA-I was therapeutic against established tumors. This mini-review discusses the emerging role of apoA-I in tumor biology and its potential as cancer therapeutic.

## Introduction

Apolipoprotein A-I (ApoA-I), the major protein component of high-density lipoprotein (HDL), is synthesized predominantly in the liver and the small intestine, and exists transiently in lipid-poor form. ApoA-I initiates assembly of HDL particles through interaction with ATP-binding cassette transporters at the surface of cells in the periphery extracting cholesterol and phospholipids. The HDL particle is further matured by lecithin cholesterol acyltransferase (LCAT) binding to apoA-I on HDL and converting cholesterol to cholesteryl ester (Sorci-Thomas et al., 2009). Cholesterol is transported within the constantly evolving ‘HDL cargo’ in circulation for excretion in liver through scavenger receptor class B, type 1 (SR-B1), a process known as reverse cholesterol transport (RCT; reviewed in Fisher et al. (2012), Rosenson et al. (2012), and Rader and Hovingh (2014)). Cholesterol accumulation and the ensuing inflammation that develops within the arterial wall are major instigators of atherosclerosis and cardiovascular disease (CVD; Hansson and Libby, 2006; Libby et al., 2009), and RCT is considered the primary atheroprotective function of HDL. A number of non-RCT atheroprotective functions of apoA-I/HDL have been described and include anti-inflammatory (Cockerill et al., 1995; Bhattacharyya et al., 2008; Rye et al., 2009; de Souza et al., 2010; Fuhrman et al., 2010; Camont et al., 2011; Gordon et al., 2011), anti-apoptotic (de Souza et al., 2010; Fuhrman et al., 2010), and anti-oxidant activities (Bhattacharyya et al., 2008). Chronic inflammation, oxidative stress, lipids, and cholesterol, which promote atherosclerosis and CVD, have all been associated with tumorigenesis (Lu et al., 2006; Freed-Pastor et al., 2012; Coussens et al., 2013; Noguti et al., 2013; Kobayashi et al., 2015). Given HDL’s beneficial role against these atherogenic processes, it was somewhat intuitive to suggest that HDL may also be protective against cancer.

## ApoA-I/Hdl Is Anti-Tumorigenic

### Clinical Observational Studies Suggest a Protective Role for ApoA-I in Cancer

Analysis of Finnish male smokers in the Alpha-Tocopherol Beta-carotene (ATBC) lung cancer prevention study identified a significant inverse association between HDL-C levels and the risk for cancer of the lung, prostate, liver, and hematopoietic system (Ahn et al., 2009). This observation was further strengthened by a large *meta*-analysis of randomized controlled trials of lipid-altering therapies suggesting an inverse relationship between plasma HDL cholesterol levels and the incidence of cancer development during the conduct of the trials (Jafri et al., 2010). Specifically, for every 10 mg/dL increase in plasma HDL cholesterol level, a significant 36% lower risk of cancer incidence was noted over 625,000 person-years of follow-up and >8,000 incidents of cancers cumulatively among the trials included in the *meta*-analysis (Jafri et al., 2010). Consistent with this finding, apoA-I has been identified as a biomarker with reduced plasma levels in patients with early stage ovarian cancer (OC) compared with normal individuals (Kozak et al., 2003, 2005; Zhang et al., 2004; Moore et al., 2006; Clarke et al., 2011; Kim et al., 2012). Furthermore, higher *apoA1* mRNA levels in pre-chemotherapy effusions from patients diagnosed with advanced stage OC was observed to be an independent prognostic marker of longer overall survival (Tuft Stavnes et al., 2014). ApoA-I together with transthyretin and transferrin, both HDL-associated proteins, β2-microglobulin, and prealbumin along with CA125 are currently being used in a US Food and drug administration approved plasma test for OC, and these combined biomarkers test is known as OVA1^®^. This test is used to identify patients with early stage OC (Kozak et al., 2005; Su et al., 2007; Nossov et al., 2008; Nosov et al., 2009). A correlation with apoA-I levels and risk of disease has also been observed in other cancers. Serum apoA-I levels were found to be twofold lower in patients undergoing surgery for pancreatic cancer compared with healthy controls (Ehmann et al., 2007). Lower serum apoA-I levels were also associated with higher risk of breast cancer (BC; Chang et al., 2007) as well as BC recurrence (Lane et al., 1995), although other studies examining BC showed either no correlation with HDL-C (Furberg et al., 2004) or an inverse association observed only in pre-menopausal women, but not in post-menopausal women (Moorman et al., 1998; Kucharska-Newton et al., 2008). In contrast, a recent study reported a positive association between serum HDL-C and apoA-I levels with BC risk; however, this direct association was only seen in women who had not used hormone replacement therapy (HRT; Martin et al., 2015). In metastatic nasopharyngeal carcinoma (NPC), higher levels of serum apoA-I measured prior to chemotherapy correlated with better overall survival (Jiang et al., 2014). Additionally, in a large European prospective study, the European Prospective Investigation into Cancer and Nutrition (EPIC) study, an inverse association was found between HDL-C levels and endometrial cancer risk (Cust et al., 2007), and similarly plasma concentrations of HDL-C and apoA-I were found to be inversely associated with the risk of colon cancer (van Duijnhoven et al., 2011). **Table 1** lists clinical studies which established an association between plasma levels of HDL-C/apoA-I and risk of developing a broad spectrum of cancers.

**Table 1:** Clinical studies investigating role of apoA-I/HDL in cancer.

Cancer type	Clinical Study Objective	Conclusion	Reference
Broad variety	Examined the relationship between serum high-density lipoprotein-cholesterol (HDL-C) and risk of overall and site-specific cancers among 29,093 Finnish male smokers in the Alpha-Tocopherol Beta-Carotene (ATBC) study cohort	Inverse association between HDL-C levels and the risk of cancer of the lung, prostate, liver, and hematopoietic system	Ahn et al., 2009
Broad variety	Analysis of 24 randomized controlled trials of lipid- altering therapy (145,743 persons) to correlate baseline and on-treatment HDL-C levels to risk of developing cancer (2.7–5.2 years follow up; 625,477 person-years)	For every 10 mg/dL increase in HDL-C, 36 % lower risk of developing cancer	Jafri et al., 2010
Ovarian	ProteinChip biomarker System and Mass spectrometry-based proteomic profiling (SELDI-TOF-MS) to identify disease associated biomarkers in serum samples from patients with ovarian cancer (OC), benign tumors, and healthy donors (109 OC, 19 benign tumors, and 56 healthy controls)	Three panels of proteins for: (i) early diagnosis of neoplasia (benign or malignant) and (ii) distinguishing benign from malignant	Kozak et al., 2003
Ovarian/breast/colon/prostate	ProteinChip biomarker System and Mass spectrometry- based proteomic profiling (SELDI-TOF-MS) and immunoassays to identify and validate biomarkers in serum samples from patients with early stage OC as compared with healthy individuals and other cancers (195 OC, 166 benign tumors, 142 healthy controls for initial screening followed by 41 OC, 20 each breast, colon, and prostate cancers with 41 healthy controls for validation by immunoassays)	Apolipoprotein A-I (ApoA-I; down-regulated in OC), truncated transthyretin (TT; down-regulated in OC), and a cleavage fragment of inter-α-trypsin inhibitor heavy chain H4 (up-regulated in OC) identified as biomarkers for OC	Zhang et al., 2004
Ovarian	Use of LC-MS/MS followed by immunoassays to identify 5 serum protein biomarkers previously reported (Kozak et al., 2003). New analysis of sera from 43 OC patients and 31 healthy controls	ApoA-I (down-regulated), TT (down-regulated), transferrin (down-regulated), and hemoglobin (up-regulated) identified as biomarkers for OC	Kozak et al., 2005
Ovarian	Independent evaluation of ApoA-I as biomarker for OC in 182 patient (42 OC, 65 benign tumors, and 76 with digestive diseases) sera collected at Mayo Clinic (1980–1989)	ApoA-I and TT were confirmed as biomarkers for OC with their expression reduced in disease	Moore et al., 2006
Ovarian	Pre-surgery blood samples (41 early stage (I/II), 51 late stage (III/IV), 40 benign disease, and 99 healthy controls) analyzed by proteomics for seven previously identified biomarkers	ApoA-I as well as TT, and connective tissue activating protein III (CTAPIII), were confirmed as a panel of biomarker together with CA125 with increased sensitivity for detection of early stage OC	Clarke et al., 2011
Ovarian	Development of multiplexed bead-based immunoassay for detection of known serum biomarkers of cancer (118 OC, 84 benign ovarian disease, 61 healthy controls)	Combination of transthyretin, and apoA-I with CA125 improved sensitivity and specificity of OC diagnosis	Kim et al., 2012
Ovarian and breast	Evaluation of apoA-I and GPX3 transcript level by qPCR in effusions, and solid tumors from patients with OC versus those with breast cancer (BC) as diagnostic tool to differentiate between these two cancers (101 OC and 20 BC effusions; 85 solid OC (43 primary, 42 metastasis))	ApoA-I transcript in all anatomic sites was higher in OC compared with BC. Higher apoA-I mRNA levels in primary diagnosis pre-chemotherapy effusions were significantly related to better overall survival	Tuft Stavnes et al., 2014
Pancreatic	ProteinChip biomarker System and Mass spectrometry- based proteomic profiling (SELDI-TOF-MS) to identify biomarkers in patient pre-surgery sera (96 cancer patients, 96 healthy controls)	ApoA-I, apoA-II and transthyretin identified as biomarkers with inverse correlation to pancreatic cancer	Ehmann et al., 2007
Breast	Lipid profile analysis in fasting patient sera prior to diagnostic biopsies (50 malignant and 50 benign)	Lower apoA-I levels predict cancer recurrence	Lane et al., 1995
Breast	Lipid profile analysis of pre-diagnostic sera from age-matched 200 (100 before age 50 and 100 at age 50 or older) case-control BC patients nested from an original cohort of 95,000 women to examine prospective association of plasma HDL-C and BC incidence	Low plasma HDL-C predicts risk of developing BC only in pre-menopausal women. Each 1 mg/dL increase in HDL-C is associated with a 4% reduction in risk of BC	Moorman et al., 1998
Breast	Assessed risk of BC associated with serum HDL-C in 38,823 Norwegian women with a median follow-up of 17.2 years	Low HDL-C, as part of the metabolic syndrome, is associated with increased postmenopausal BC risk	Furberg et al., 2004
Breast	Lipid profile analysis in fasting patient (Taiwanese) sera (150 cancer and 71 healthy controls) and association with BC risk	ApoA-I levels in serum inversely associated with BC	Chang et al., 2007
Breast	Evaluate association of baseline HDL-C levels with cancer incidence using data from the Atherosclerosis Risk in Communities Study (ARIC) cohort with follow-up from 1987 through 2000	Modest direct association of low HDL-C with risk of developing BC only in women who were premenopausal at baseline	Kucharska-Newton et al., 2008
Breast	Multiple time point measurements of serum lipids and lipoproteins in a nested case-control (279 cases and 558 matched control subjects) study within a randomized long-term dietary intervention trial with 4,690 women for an average of 10 years to assess the association of serum lipids with the risk of cancer incidence based on menopausal status and use of hormone replacement therapy (HRT)	HDL-C and apoA-I were positively associated with BC risk only when HRT was not used	Martin et al., 2015

Nasopharyngeal	Retrospective analysis of 807 patients with metastatic nasopharyngeal carcinoma (NPC) to assess prognostic value of baseline serum lipids in predicting overall survival	Higher values of HDL-C and apoA-I were associated with improved overall survival	Jiang et al., 2014
Endometrial	Examined the association of pre-diagnostic plasma levels of lipids, lipoproteins and other metabolic factors in developing cancer in a nested case-control (262 cases and 546 matched control subjects) study of the European Prospective Investigation into Cancer and Nutrition (EPIC; 520,000 participants from 10 western European countries)	HDL-C levels were inversely correlated with the risk of developing cancer	Cust et al., 2007
Colorectal	Examined the association of pre-diagnostic plasma levels of lipids, lipoproteins and other metabolic factors in developing cancer in a nested case-control (1,238 cases, 1,238 matched control subjects) study of the European Prospective Investigation into Cancer and Nutrition (EPIC; 520,000 participants from 10 western European countries)	Higher pre-diagnostic HDL-C and apoA-I were statistically significantly inversely associated with risk of colon cancer, but not rectal cancer	van Duijnhoven et al., 2011


*Clinical studies which established an association between plasma levels of apoA-I/HDL-C and risk of developing a broad spectrum of cancers.*

### ApoA-I is Protective in Mouse Tumor Models

Although the above-described clinical studies suggested, for the most part, lower apoA-I plasma levels correlate with poorer patient outcomes in a variety of cancers, they fall short of inferring causality. The first evidence supporting the notion that increasing levels of apoA-I may be protective against tumor development came from mouse tumor studies. Accordingly, there was an apoA-I gene dosage effect on Lewis lung tumor growth, with tumor size smallest for mice homozygous for the human *apoA1* transgene (h*A-I Tg*^+/+^), intermediate tumor size in mice heterozygous for the human *apoA1* transgene (h*A-I Tg*^+/-^, referred to hereon as simply *apoA1* transgenic (*A-I Tg*)), and largest tumor size in *apoA1* null mice (*A-I* KO; Zamanian-Daryoush et al., 2013). Furthermore, overall tumor burden and metastasis was strongly suppressed in response to challenge with the aggressive and metastatic syngeneic melanoma cells (B16F10L) in animals expressing the *apoA1* transgene relative to *apoA1* null or wild-type (WT) animals, culminating in improved overall survival (Zamanian-Daryoush et al., 2013). Similarly, in a mouse model of OC (ID8), *ApoA-I* transgene expression reduced tumor burden and led to a significant increase in survival (Su et al., 2010).

### Infusion of Human ApoA-I Inhibits Tumor Development and is Therapeutic against Established Mouse Tumors

Unequivocal support of an anti-neoplastic role for apoA-I came from animal studies where subcutaneous injections of human apoA-I prior to tumor inoculation prevented tumor development in *A-I* KO mice (Zamanian-Daryoush et al., 2013). More importantly, in a physiologically relevant setting, provision of apoA-I after tumor establishment not only prevented further development of the tumor but also led to tumor shrinkage (Zamanian-Daryoush et al., 2013). Although these protective effects were seen with the syngeneic mouse melanoma tumor, B16F10L, apoA-I therapy was also shown to be effective against human melanoma (A375) in nude mice (Zamanian-Daryoush et al., 2013). Based on these observations a potential role for apoA-I/HDL as an anti-cancer therapeutic has been proposed (Zamanian-Daryoush et al., 2013).

## ApoA-I/Hdl Mimetic Peptides Exhibit Anti-Tumor Activity

There has been considerable interest, in the HDL field, surrounding the therapeutic potential of a series of 18 amino acid peptides known as apoA-I/HDL mimetics, against cardiovascular disease. These short peptides have certain functional properties of the repeating amphipathic α-helices of apoA-I without actually sharing any sequence homology (Getz and Reardon, 2014; Reddy et al., 2014). These peptides were originally screened for their ability to form class A amphipathic helices with lipid binding capability, a salient functional feature of apoA-I (Kanellis et al., 1980; Segrest et al., 1983; Anantharamaiah et al., 1985; Mendez et al., 1994). ApoA-I/HDL mimetics have been successfully used in a number of mouse models of atherosclerosis (Garber et al., 2001; Navab et al., 2010; Getz and Reardon, 2011) and shown to have anti-inflammatory and anti-oxidant activities as well as the ability to promote RCT (D’Souza et al., 2010; Ditiatkovski et al., 2013). These peptides have similar binding affinities for non-oxidized lipids but a significantly higher affinity for pro-inflammatory oxidized lipids (Van Lenten et al., 2008) and for lysophosphatidic acid (LPA; Su et al., 2010), relative to full-length apoA-I. LPA is known to promote tumor development (Mills and Moolenaar, 2003; Li et al., 2009) and has been identified as a biomarker for OC (Xu et al., 1998; Sutphen et al., 2004). Consequently, due to their anti-inflammatory activity and increased lipid binding properties apoA-I/HDL mimetics, when tested for anti-tumor activity, were shown to have protective activity in multiple mouse tumor models including ovarian (Su et al., 2010; Gao et al., 2011, 2012; Ganapathy et al., 2012), and colon cancer (Su et al., 2012). It was shown for OC that the primary functional activity of the mimetic peptides was due to squelching of the bioactive, tumor-promoting LPA and oxidized lipids that serve as potent tumor growth and angiogenic factors. The mimetic peptides 4F and 5F when injected or administered orally, inhibited tumor growth and reduced plasma levels of LPA in the tumor bearing mice (Su et al., 2010). Likewise, in another study the functional target of the apoA-I/HDL mimetic peptide L-4F was presumed to be LPA as administration of this HDL-mimetic peptide was shown to decrease circulating levels of LPA in mimetic-treated animals and demonstrated protection against colon cancer (Su et al., 2012). ApoA-I mimetics were also reported to inhibit tumor angiogenesis *in vivo* and abrogate growth factor induced proliferation, migration, invasion, and tube formation of endothelial cells *in vitro* (Gao et al., 2011). The mimetics significantly decreased LPA-induced vascular endothelial growth factor (VEGF) production by cancer cells through inhibition of hypoxia-inducible factor-1α (HIF-1α) as well as interfering with VEGF-induced signaling in endothelial cells thus mitigating VEGF’s ability to promote angiogenesis (Gao et al., 2011, 2012). Furthermore, the mimetics were shown to inhibit cell viability and proliferation of OC cells by reducing the oxidative stress in cancer cells through induced expression of the tumor suppressor enzyme, manganese-containing superoxide dismutase (MnSOD; Ganapathy et al., 2012). Although these apoA-I/HDL peptide mimetics have demonstrated very interesting anti-tumor properties, their apparent mechanism of action (squelching LPA for the most part) does not appear to coincide with the anti-tumor mechanisms of actual apoA-I/HDL (Zamanian-Daryoush et al., 2013).

## Mechanism Of ApoA-I/Hdl Anti-Tumor Activity

### ApoA-I/HDL Negatively Impacts Tumor-permissive Features of the Tumor Microenvironment

**FIGURE 1:**
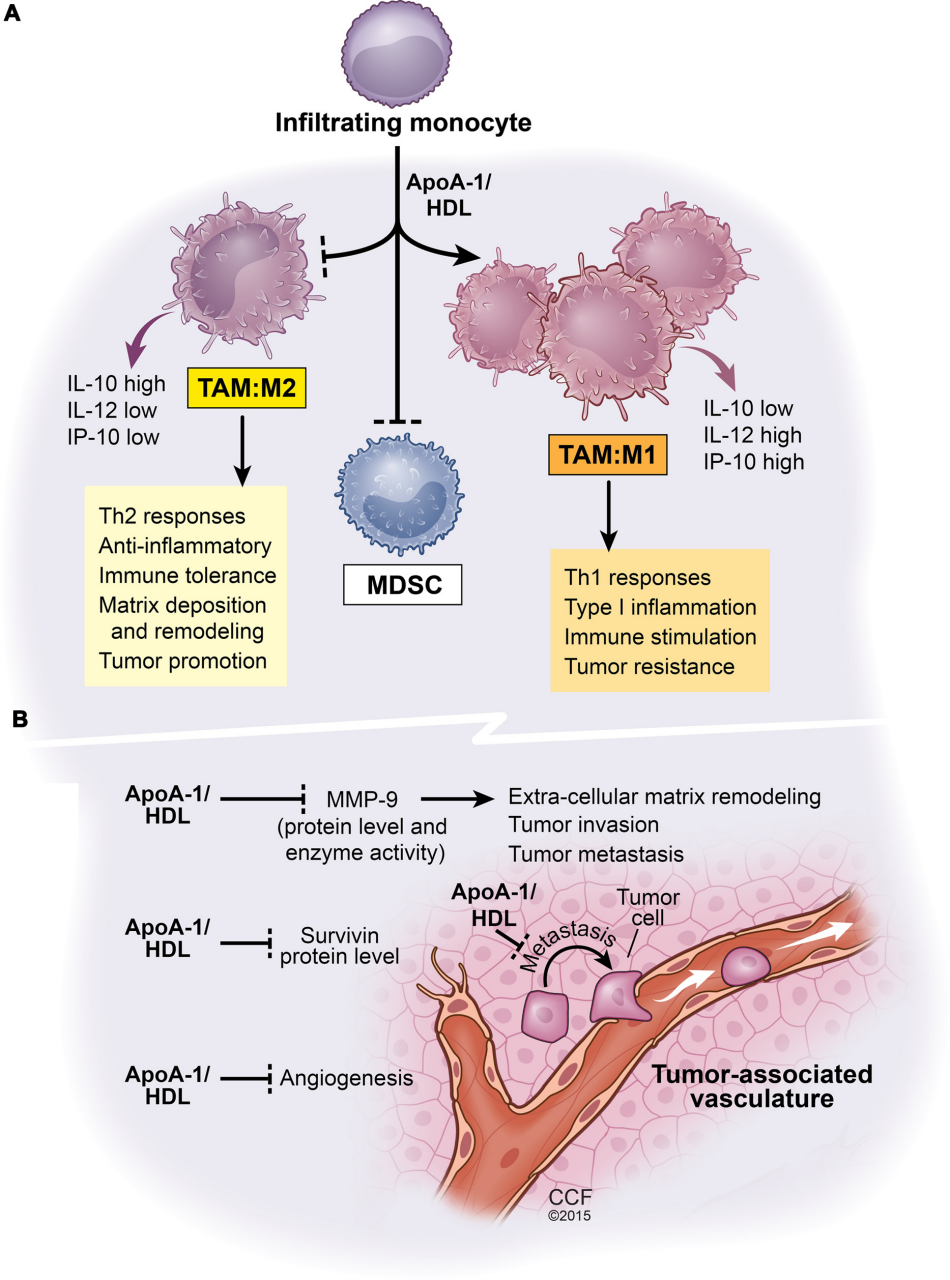
**Net functional effects of apoA1/HDL in the tumor microenvironment.** This scheme is based on comparative analyses of B16F10L melanoma tumors resected from mice either deficient in apoA-I or mice expressing human apoA-I (Zamanian-Daryoush et al., 2013). **(A)** The effect of apoA-I/HDL on tumor-infiltrating myeloid cell population. ApoA-I/HDL promotes the accumulation of TAMs with an M1-like anti-tumor phenotype and inhibits the accumulation of the tumor promoting M2-like TAMS as well as MDSCs. **(B)** The impact of apoA-I/HDL on tumor vasculature, metastasis, and tumor survival, all phenomena that are subject to regulation by myeloid cells. Apolipoprotein A-I, ApoA-I; high density lipoprotein, HDL; interleukin-10, IL-10; interleukin-12, IL-12; tumor-associated macrophages, TAM; myeloid-derived suppressor cells, MDSCs; matrix metalloproteinase-9, MMP-9. Solid lines ending in arrow head signify promotion whereas solid lines ending with a perpendicular dotted line signify inhibition of biological pathways.

Although the exact molecular mechanism of apoA-I/HDL anti-tumor activity is not known, studies with the syngeneic B16F10L tumors comparing those from *apoA1* transgenic vs. *A-I* KO mice revealed that the overall net impact of host apoA-I on the tumor microenvironment is profound and manifold. These include but are not limited to the following as depicted in **Figure 1**: decreased recruitment of myeloid-derived suppressor cells (MDSC) and reduced angiogenesis resulting in decreased tumor volume; decreased matrix metalloproteinase-9 (MMP9) protein levels and enzyme activity; decreased overall metastasis, increased accumulation of tumor-associated macrophages (TAMs) with an M1-like anti-tumor phenotype; increased levels of tumor cell killing macrophages; increased recruitment of CD8T cells and decreased levels of the anti-apoptotic protein survivin within the tumor bed (Zamanian-Daryoush et al., 2013). Unexpectedly, levels of the pro-angiogenic protein, VEGF-A, was higher in tumors from *A-I Tg* mice relative to *A1*KO mice (Zamanian-Daryoush et al., 2013). It may be that the VEGF-A protein quantified in these studies was VEGF-Ax, an anti-angiogenic VEGF-A variant recently reported to be a product of translational read-through (Eswarappa et al., 2014) though this remains to be proven. Both the antibody and the oligonucleotide probe sets used to detect VEGF-A in Zamanian-Daryoush et al. (2013) cannot distinguish between the two forms. Unlike the observations with apoA-I/HDL peptide mimetics, which appear to inhibit tumor growth by squelching/decreasing circulating levels of the bioactive tumor promoting lipid LPA; the plasma level of LPA, was found to be similar in B16F10L melanoma tumor-bearing *A-I* KO vs. *A-I Tg* animals (Zamanian-Daryoush et al., 2013), thus eliminating titration of this bioactive lipid as a functional mechanism, in this setting, for apoA-I protein (Zamanian-Daryoush et al., 2013).

### ApoA-I/HDL Anti-tumor Activity Requires an Intact Innate and Adaptive Immune System for Full Anti-tumor Activity

ApoA-I infusion studies with mice deficient in various aspects of immune system revealed that complete apoA-I anti-tumor activity requires both innate and adaptive arms of immunity. In particular, TAMs isolated from tumors resected from *A-I Tg* animals were skewed toward an M1-like phenotype and macrophages isolated from tumor-bearing *A-I Tg* animals exhibited enhanced cytotoxicity toward B16F10L tumor cells *in vitro* (Zamanian-Daryoush et al., 2013). Macrophages are innate immune cells with pivotal roles in tumor biology. Their ability to destroy tumors or promote their development is a function of their phenotypic plasticity, which is governed, in major part, by growth factors, cytokines, and bioactive lipids present in the tumor microenvironment (Mantovani et al., 2005; Martinez et al., 2009; Solinas et al., 2009; Liu and Yang, 2013). TAMs are in general, alternatively activated (M2-like phenotype) and promote angiogenesis, tumor survival, and metastasis, whereas classically activated macrophages (M1-like phenotype) inhibit these processes leading to tumor inhibition. Conversion of macrophage phenotype from M2 to M1 has been demonstrated to result in tumor inhibition in several murine tumor models (Colombo and Mantovani, 2005; Guiducci et al., 2005). The finding that in the tumor microenvironment, apoA-I/HDL promotes the accumulation of macrophages with pro-inflammatory, classically activated anti-tumor M1-like phenotype is in direct contrast with the traditional anti-inflammatory and immunosuppressive functions described for HDL in a typical inflammatory setting (Murphy et al., 2012). How apoA-I/HDL triggers this uncharacteristic effect is not known but is currently under investigation. The transcription factor activator of transcription factor 3 (ATF3) was recently identified as mediating anti-inflammatory and immune modulatory functions of HDL in macrophages (De Nardo et al., 2014). HDL induced the transcriptional expression of ATF3, which in turn inhibited the expression of some 130 toll-like receptor (TLR)-mediated pro-inflammatory genes with immune response-related functions (De Nardo et al., 2014). TLRs recognize pathogen-associated molecular pattern (PAMPs) molecules from viruses and microorganisms along with endogenously derived ligands such as damage-associated molecular pattern molecules (DAMPs) and can play important roles in both pro-and anti-tumor responses (Pradere et al., 2014). HDL inhibits lipopolysaccharide (LPS)-mediated TLR4 signaling by squelching the ligand itself and although HDL pretreatment was found to decrease cellular cholesterol concentration, it did not inhibit CpG-induced TLR9 signaling *per se* but reduced downstream gene expression by CpG through induction of ATF3 (De Nardo et al., 2014). These results indicate that apoA1/HDL can influence TLR signaling in normal inflammatory settings via at least two different routes.

HDL is an integral component of host immunity because its cargo, a series of bioactive proteins and lipids, has immunomodulatory activities. These activities include but are not limited to (i) ability to scavenge bacterial outer membrane components, LPS(Gram-negative bacteria) or lipoteichoic acid (LTA; Gram-positive bacteria) limiting their pro-inflammatory toxicity and protecting against sepsis (Levine et al., 1993; Birjmohun et al., 2007; Wendel et al., 2007; Guo et al., 2013), (ii) protection against intracellular bacteria such as mycobacteria (Cruz et al., 2008) and parasites (Vanhollebeke and Pays, 2010), (iii) in concert with ATP-binding cassette transporters ABCA1 and ABCG1, HDL has a role in control of hematopoietic stem and multipotential progenitor cell proliferation (Yvan-Charvet et al., 2010), and (iv) influencing immune cell response by modulating cholesterol content in membrane lipid rafts. Cholesterol accumulation in immune cells increases signaling by stabilizing lipid rafts in the plasma membrane as well as in other cellular membranes. Lipid rafts are cholesterol enriched micro-domains with sphingolipids and serve as docking sites for several receptors with important immunological functions, including TLRs (Fessler and Parks, 2011) and T- and B-cell receptors (TCRs; Gupta and DeFranco, 2007; Kabouridis and Jury, 2008). Cholesterol eﬄux by apoA-I/HDL via ABCA1 and ABCG1 disrupts lipid rafts and their associated signaling pathways (Triantafilou et al., 2002; Zhu et al., 2010). Thus cholesterol eﬄux-mediated modification of immune response was noted in: (i) monocytes/macrophages, inhibiting their activation and recruitment (Murphy et al., 2008) as well as skewing macrophages toward an M2-like anti-inflammatory immunosuppressive phenotype (Smythies et al., 2010; Feig et al., 2011; Murphy et al., 2012); (ii) neutrophils, inhibiting their migration and adhesion (Murphy et al., 2011); (iii) dendritic cells (DCs), inhibiting their maturation and their ability to induce T cell activation (Perrin-Cocon et al., 2012; Wang et al., 2012), while the immunoactive lipid sphingosine 1-phosphate (S1P) carried in HDL promotes an anti-inflammatory phenotype in DCs thus inhibiting a Th1 response (Idzko et al., 2002); (iv) T cells, inhibiting their activation and proliferation in peripheral lymph nodes in mice fed a high fat diet (Wilhelm et al., 2009). These observations are clearly supportive of apo A-I/HDL’s role as a physiological modulator of membrane cholesterol and immune function in different pathophysiological conditions.

### Deletion of ApoA-I/HDL Receptors Lead to Altered Macrophage Phenotypes

Recently, myeloid-specific or global deletion of *Abcg1* in mice was reported to be anti-tumorigenic with macrophages displaying a clear shift from an M2-like to an M1-like phenotype (Sag et al., 2015). The anti-tumor effect was demonstrated to be driven by myeloid cells, and in large part due to the increased accumulation of cholesterol in macrophages. Studies on bone marrow-derived macrophages (BMDMs) from *Abcg1*^-^*^/^*^-^ mice compared to WT mice after treatment with either M1 or M2 polarizing cytokines and comparative analysis of M1 or M2 cell markers showed that the *Abcg1*^-^*^/^*^-^ BMDMs were intrinsically biased to an M1 pro-inflammatory phenotype (Sag et al., 2015). These findings underscore the significance of cholesterol homeostasis in tumor immunity. Loss of *Abca1* in myeloid cells results in increased accumulation of free cholesterol and enhanced pro-inflammatory responses in macrophages, highlighting the ormal anti-inflammatory function of these transporters (Yvan-Charvet et al., 2008; Zhu et al., 2008; Tang et al., 2009). Correspondingly, macrophages from mice globally deleted for scavenger receptor class B, type1 (*Sr-b1*), have also been shown to exhibit hyperinflammatory responses (Cai et al., 2012). However, unlike the situation with *Abca1* or *Abcg1* deletion, the heightened inflammatory responses are not due to alterations in cell membrane or total cell cholesterol levels (Cai et al., 2012). It is not known what effect loss of ABCA1 or SR-B1 protein expression either globally or myeloid-specifically has on tumorigenesis but by extrapolation from tumor studies in mice with ABCG1 loss, one might predict a protective effect due to the heightened pro-inflammatory phenotype these transporter-/receptor-deleted macrophages are known to exhibit. The emerging paradigm from the studies by Zamanian-Daryoush and that of Sag et al., is that TAMs can be converted from an M2-like to an M1-like phenotype by either a hard-wiring to an M1 anti-tumor phenotype through loss of cholesterol/phospholipid transporter ABCG1 (Sag et al., 2015) and perhaps ABCA1 and SR-B1 or in response to increased apoA1/HDL levels; which by unknown mechanisms alters immune responses to factors within the tumor microenvironment and promotes the accumulation of TAMs with an M1-like phenotype at the expense of TAMs with an immune suppressive M2-like phenotype.

## Conclusion

In cancer, lipid and cholesterol homeostasis is often dysregulated to facilitate the cancer cells’ increased demand for these building blocks which are required for proliferation and evasion of apoptosis (Schaffner, 1981; Yoshioka et al., 2000; Kolanjiappan et al., 2003; Platz et al., 2009; Smith and Land, 2012). To this end, tumor cells can manipulate their intracellular cholesterol level by reducing expression of ABCA1 which eﬄuxes cholesterol and increasing the expression of SR-B1 which influxes cholesterol. This phenomenon has been reported in several prostate, colon and BCs (Cao et al., 2004; Mooberry et al., 2010; Su et al., 2010; Smith and Land, 2012; Lee et al., 2013).

The ability to modulate lipid and cholesterol movement is at the core of apoA-I/HDL’s actions resulting in profound physiological and cell phenotypic effects. Changes in cholesterol metabolism or levels of components of cholesterol homeostasis namely apoA-I/HDL, ABCA1, ABCG1, and SR-B1 are known to affect immune responses which in turn impact anti-tumor effects. In fact, apoA-I/HDL’s anti-tumor effects were observed maximally only in fully immune-competent animals (Zamanian-Daryoush et al., 2013). Anti-tumor effects of apoA-I/HDL could be related to (i) the ability of apoA-I/HDL to modulate cholesterol content in immune or tumor cell membrane lipid rafts thus influencing signaling pathways, (ii) the lipid rafts’ role as a platform for biologically active lipids and proteins that may impact the immune response, (iii) the cross-talk between the tumor and surrounding stromal cells. These possibilities are not mutually exclusive. ApoA-I/HDL peptide mimetics appear to primarily function through titrating out bioactive lipids and molecules which function as potent tumor cell angiogenic and growth factors. Although apoA-I/HDL may also perform similar titrating actions, the extent to which this activity is consequential in cancers other than in ovarian and perhaps colon cancer, is not as apparent. ApoA-I/HDL appears to function as an anti-tumor agent in large part by modulating the anti-tumor immune response. In the context of infection or atherogenesis, apoA-I/HDL modulates macrophages toward an anti-inflammatory M2-like phenotype by eﬄuxing cholesterol but in the tumor microenvironment, apoA-I/HDL promotes the accumulation of M1-like macrophages. At the present time, we do not know the mechanism involved in this process but this apparent dichotomy of apoA-I/HDL functional response in different inflammatory settings underscores the complexity of apoA-I/HDL biology and poses intellectual and experimental challenges toward a better understanding of this multifaceted plasma component.

## Conflict of Interest Statement

Drs. Maryam Zamanian-Daryoush and Joseph A. DiDonato report being listed as co-inventor on pending and issued patents held by the Cleveland Clinic.
